# Thermomechanical Behavior and Mesoscale Simulation of Conglomerate under Impact Loading for Geothermal Engineering

**DOI:** 10.1021/acsomega.5c10038

**Published:** 2026-01-02

**Authors:** Wei Cheng, Huifeng He, Chengfu Han, Lei Chen, Liang Zhu, Yishan Lou

**Affiliations:** † School of Petroleum Engineering, National Engineering Research Center for Oil & Gas Drilling and Completion Technology, 643370 Yangtze University, Wuhan 430100, China; ‡ School of Intelligent Manufacturing, Jingchu Institute of Technology, Jingmen 448000, China; § Changqing Drilling Corporation, Sichuan Petroleum Drilling Engineering Company, Xi’an 710018, China

## Abstract

To investigate the mechanical response of conglomerate under coupled high-temperature and dynamic loading conditions, impact compression tests were conducted using a ⌀50 mm Split Hopkinson Pressure Bar (SHPB) system at temperatures of 25 °C, 100 °C, 150 °C, 200 °C, 250 °C, and 300 °C. At lower temperatures, the specimens exhibit greater energy absorption and dissipation capacity, which promotes crack propagation and energy release. In contrast, at elevated temperatures, energy reflection becomes more pronounced while dissipation decreases, leading to a more brittle failure mode. Mesoscopic analysis based on finite element simulations further clarifies the influence of temperature on stress distribution and crack evolution during impact failure. The results indicate that the dynamic compressive strength of conglomerate decreases with increasing temperature, with the peak stress dropping from 192 MPa at 25 °C to 116 MPa at 300 °C, corresponding to a reduction of approximately 39.5%. Fractal dimension analysis reveals a positive correlation between fragmentation degree and temperature, with significantly finer fragments observed at 300 °C compared to 25 °C. These findings provide theoretical insights and practical guidance for evaluating the stability of high-temperature conglomerate formations and optimizing rock-breaking parameters in geothermal engineering.

## Graphical abstract

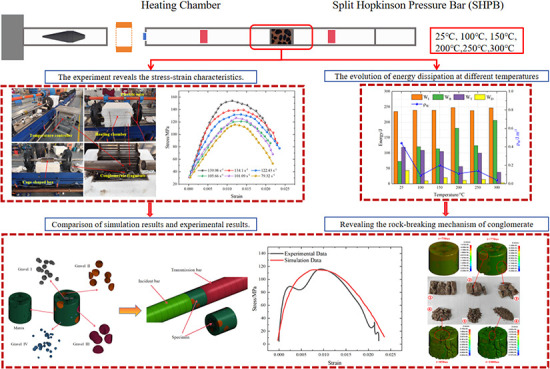

## 1 Introduction

In recent years, with the rapid expansion and increasing complexity of rock mass engineering projects, both research and practical applications in rock mechanics have advanced significantly, giving rise to a range of new scientific and engineering challenges.^1,2^ As a renewable and clean energy source, geothermal energy has become one of the most promising strategic alternatives for future development. Globally, nearly 90% of geothermal resources are categorized as hot dry rock (HDR). In China, HDR resources are widely distributed; however, most reservoirs are characterized by great burial depth, high temperatures, complex lithological conditions, and considerable challenges in exploitation.^3^ In this context, growing attention has been directed to the effects of elevated temperatures on the physical and mechanical properties of rocks, as thermal effects are known to exert a substantial influence on rock mechanical behavior.^4–9^ Extensive investigations have been conducted on the static failure characteristics of rocks under elevated temperature conditions. For example, Huang et al.^10^ performed uniaxial compression and Brazilian splitting tests on granite after high-temperature treatment and explored the variations in uniaxial compressive strength, elastic modulus, tensile strength, and the compressive-to-tensile strength ratio with temperature, elucidating the thermal degradation mechanism of granite. Hao et al.^11^ evaluated the mechanical behavior of thermally damaged sandstone using uniaxial compression and Brazilian tests. Their results indicated that the tensile strength of sandstone first decreased and then increased sharply with temperature, while the compressive strength initially declined and subsequently increased. Dwivedi et al.,^12^ motivated by the thermal conditions expected in underground nuclear waste repositories, studied a range of thermomechanical properties of Indian granite (IG) between 30 °C and 160 °C, including Young’s modulus, uniaxial compressive strength, tensile strength, Poisson’s ratio, linear thermal expansion coefficient, and creep behavior, along with microcrack evolution observed via scanning electron microscopy (SEM). Kumari et al.^13^ conducted Brazilian splitting tests on two types of Australian granite (Strathbogie and Harcourt) to assess the influence of temperature (up to 1000 °C) and different heating–cooling paths on tensile strength, and analyzed thermally induced microstructural changes.

Most of the aforementioned studies focused on conventional rock types such as sandstone, granite, limestone, and marble, whereas research on the thermal effects on conglomerate is relatively scarce. The existing studies on conglomerate are primarily concerned with its response to quasi-static loading conditions.^14,15^ For instance, Wei et al.^16,17^ performed true triaxial tests and CT scans to study the strength and failure characteristics of conglomerate under different stress conditions. They found that the failure process and strength characteristics of conglomerate are significantly influenced by the horizontal principal stress, generally displaying brittle failure, and that the strength increases linearly with the horizontal stress. Wang et al.^18^ conducted uniaxial compression tests on conglomerate and observed widespread microcrack formation around clasts. Their study also revealed that clast content is a key factor controlling the macroscopic mechanical properties of conglomerate, with higher clast content leading to reduced compressive strength and elastic modulus, but enhanced ductility. Luo et al.^19^ employed the discrete element software PFC2D to simulate uniaxial compression tests on conglomerate and analyzed the effects of bonding strength, clast content, and clast geometry on the deformation and failure behavior. Li et al.^20^ performed both uniaxial and triaxial compression tests on conglomerate samples, showing that tensile failure dominates under uniaxial conditions, while shear or fragmented flow with volumetric expansion occurs under triaxial loading.

However, in geothermal exploration and development, conglomerate formations are often subjected to impact loads during drilling operations. Such impact loading can cause rock failure within a very short time frame and results in mechanical responses that differ substantially from those observed under static loading. Therefore, it is insufficient to study conglomerate failure characteristics based solely on static tests.^21^ Recent studies have increasingly used dynamic mechanical testing and energy dissipation analysis to investigate rock deformation and failure under impact conditions. For example, Li et al.^22^ used a Split Hopkinson Pressure Bar (SHPB) system to study dynamic compression behavior and clarified the multiscale damage evolution and energy dissipation mechanisms in rock materials under different loading rates. They found that higher loading rates produced more microcracks of smaller sizes, larger numbers of macroscopic cracks, and greater energy absorption in sandstone specimens. Li et al.^23^ conducted SHPB tests on limestone, dolomite, and sandstone to obtain dynamic strength factors, dissipated energy density, and particle size distributions under varying strain rates, and further used the crystal-based discrete element method to study the high-strain-rate mechanical and damage behavior. Wu et al.^24^ investigated the influence of loading rate on the dynamic mechanical properties of sandstone under high-temperature conditions using a SHPB setup. Their results indicated a quadratic increase in peak strength with loading rate, implying a strain-rate strengthening effect. Zhu et al.^49^ employed a modified large-diameter SHPB device to carry out cyclic impact compression tests on granite and analyzed its mechanical properties and energy absorption characteristics under repeated impact loading. Asadi et al.^25–31^ performed comprehensive numerical simulations of the dynamic fracture and fragmentation behavior of rocks, supplemented by SHPB experiments to evaluate rock response under impact loading. Their work systematically examined the influence of specimen size, particle size distribution, defect geometry, and loading rate on the dynamic tensile strength, dynamic compressive strength, and fracture patterns of rock. These studies provide valuable theoretical and methodological foundations for the present work, particularly in analyzing energy dissipation, validating numerical simulation results, and further elucidating the coupled effects of temperature and dynamic loading rate on rock behavior.

Most of these studies focus on conventional rocks under dynamic loading, while relatively few have addressed the mechanical behavior of conglomerate under impact loading. Moreover, studies on the failure mechanisms of conglomerate under the coupled effects of temperature and impact loading remain rare. Given the critical importance of conglomerate stability in geothermal drilling operations, it is essential to investigate its mechanical properties under combined thermal and dynamic conditions.

In this study, high-temperature dynamic compression tests were conducted on conglomerate using a Split Hopkinson Pressure Bar (SHPB) system. The effects of temperature and impact loading on the dynamic fragmentation characteristics of conglomerate were analyzed. Fractal dimension analysis was applied to characterize the fragmentation patterns, and numerical simulations were conducted to study the dynamic failure processes under varying impact loads. The results provide theoretical insights and practical references for understanding the impact failure mechanisms of conglomerate and optimizing impact-related parameters during geothermal resource exploration.

## 2 Experimental Overview

### 2.1 Specimen Preparation

The conglomerate core samples used in this study were obtained from a block within the Junggar Basin in Xinjiang, China. Rock blocks with good integrity and homogeneity were selected as representative specimens. In accordance with the requirements for rock mechanical testing, the dimensions of the specimens for quasi-static loading tests were set to ⌀25 mm × 50 mm, based on the principles of quasi-static loading and prior research findings.^32^ For dynamic loading tests, the specimen dimensions were ⌀50 mm × 50 mm.

The specimens were processed using a TQZ-200 vertical coring machine, a YQ-250 rock cutting machine, and a DYM-2S double-end grinding machine. The parallelism and perpendicularity deviations of the specimen end faces were controlled to be less than 0.02 mm. The basic mechanical properties of the conglomerate were determined using a TAW-2000 triaxial testing system under quasi-static loading conditions. The physical and mechanical parameters of the conglomerate are listed in Table 1. Figure 1 shows the quasi-static test procedure, and Figure 2 displays the specimens prepared for dynamic loading tests.

**Table 1:** Static Load Physical and Mechanical Parameters of Conglomerate Specimens

Density/kg.m^–3^	Poisson’s ratio	Strain rate/s^–1^	Elastic modulus/GPa	Static compressive strength/MPa
2310	0.29	1e-5	21.76	112.99

**Figure 1:**
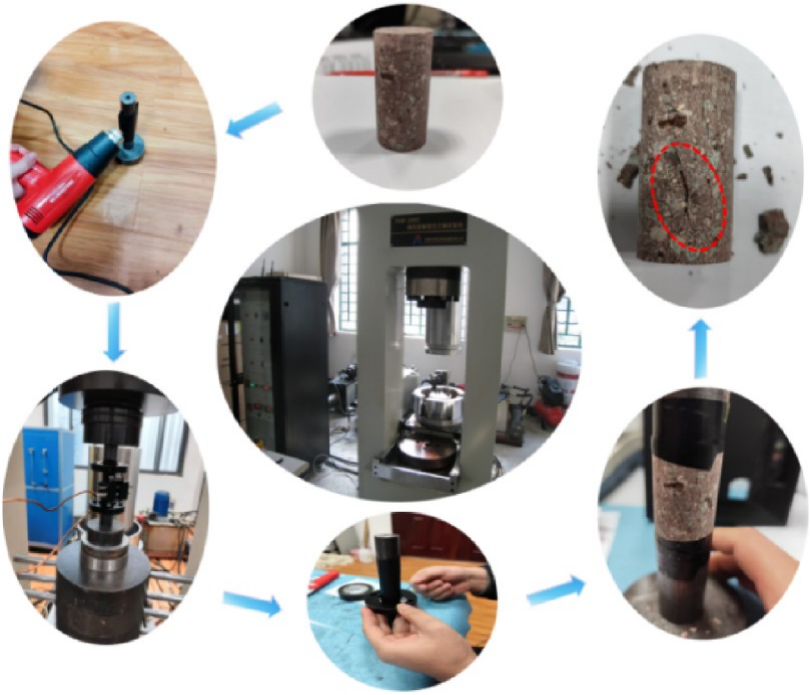
Quasi-static compression test procedure for conglomerate specimens.

**Figure 2:**
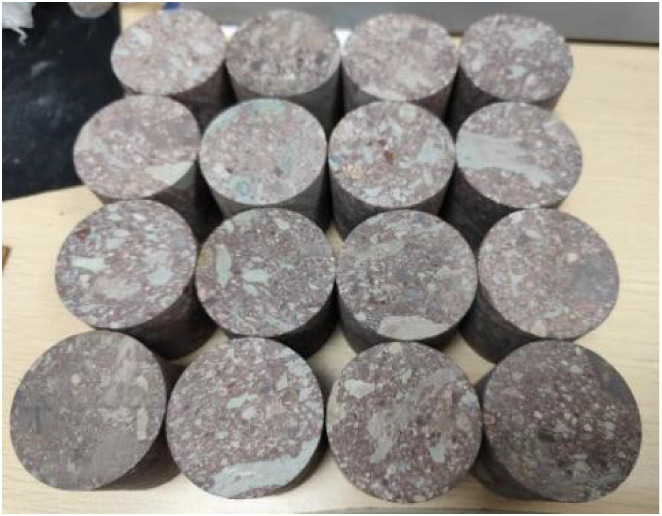
Conglomerate specimens for dynamic loading tests.

### 2.2 SHPB Test Apparatus

The impact tests were conducted using a large-diameter Split Hopkinson Pressure Bar (SHPB) apparatus (⌀50 mm) manufactured by Zhongde Electromechanical Co., Ltd., Shandong, China. A schematic of the experimental setup is shown in Figure 3. This system enables the investigation of the dynamic mechanical response of rock materials under various high strain rate impact conditions. It allows for the acquisition of stress–strain curves at different strain rates, which provides essential physical parameters for numerical simulation and analysis of the conglomerate. Additionally, high-speed imaging can be employed in conjunction with the SHPB system to analyze the deformation and failure characteristics of conglomerate under various strain rate conditions.

**Figure 3:**
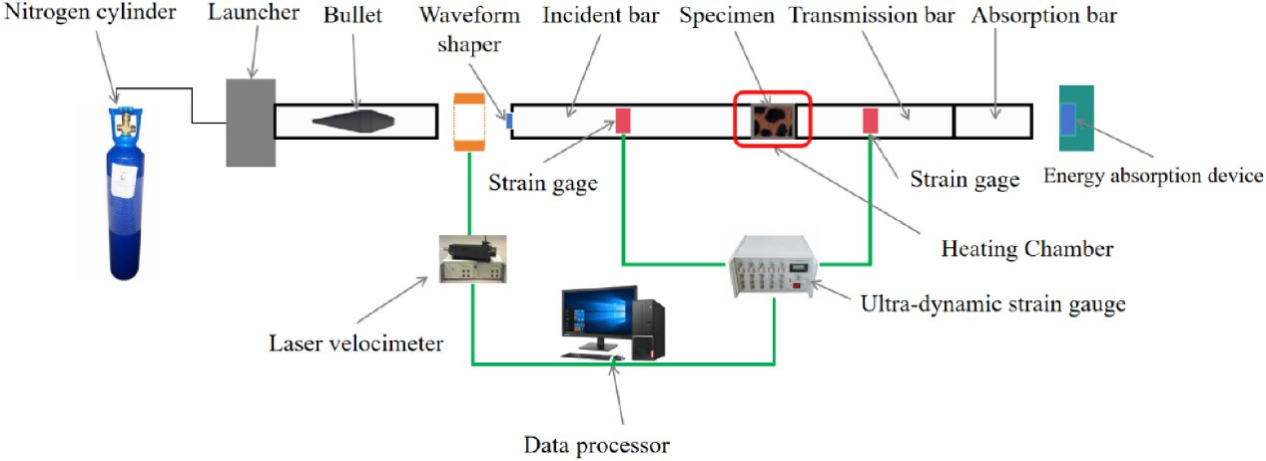
Schematic of the dynamic loading test setup for conglomerate specimens using the SHPB system.

The incident bar, transmission bar, absorption bar, and striker in the SHPB system are all made of 40Cr alloy steel, with an elastic modulus of 206 GPa, a density of 7810 kg/m^3^, and a longitudinal wave velocity of 5100 m/s. The diameter of the SHPB bars is 50 mm, with the incident and transmission bars measuring 3.5 and 3.0 m in length, respectively. Under the action of compressed air, the striker impacts the incident bar, generating a stress pulse at the bar end. A spindle-shaped striker is used to produce a half-sine stress wave, enabling loading under a constant strain rate during the test process,^33,34^ as illustrated in Figure 4.

**Figure 4:**
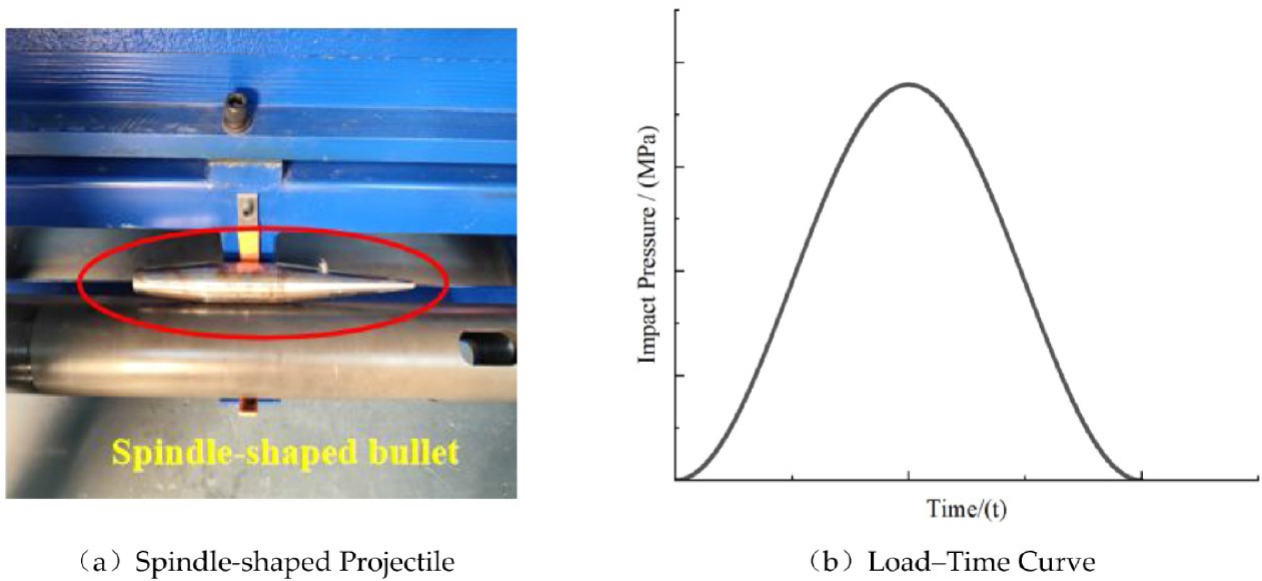
Waveform generation device. (a) Spindle-shaped projectile; (b) load waveform.

The high-temperature system utilizes a ZONE-DE model Hopkinson bar furnace, consisting primarily of a temperature control system, a lower furnace chamber, and an upper furnace chamber, as shown in Figure 5. The furnace is constructed using microporous calcium silicate boards, with high-temperature resistance wires installed inside the lower chamber. These resistance wires are connected to the temperature control system, which allows for precise temperature setting, with a maximum heating temperature of 1000 °C. The heating rate can also be set via the control system, with a maximum rate of 10 °C/min. The temperature acquisition system is equipped with sensors that continuously monitor and display the specimen temperature in real time.

**Figure 5:**
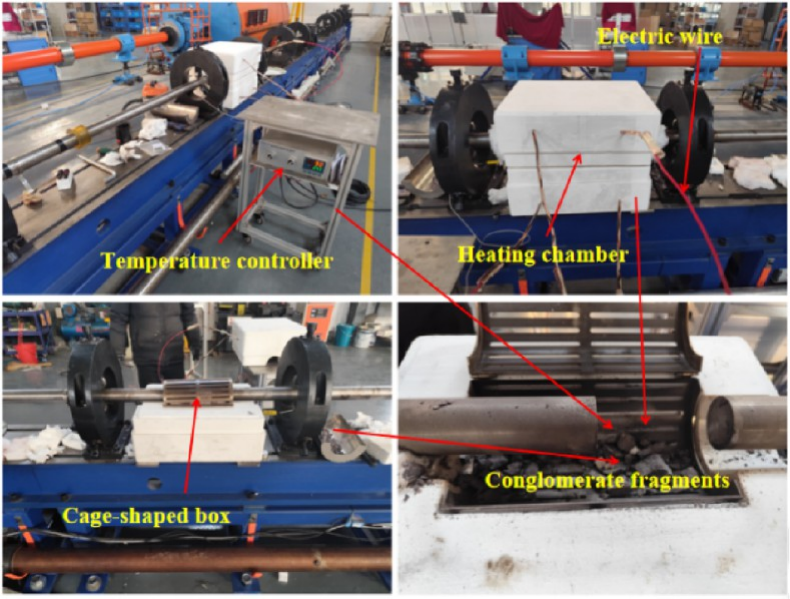
High-temperature control system.

### 2.3 Experimental Method

According to stress wave theory and the one-dimensional elastic wave assumption, if the dispersion effects of stress wave propagation in the elastic bars are neglected, the stress wave will not undergo distortion or attenuation during transmission.^35^ Upon contact with the specimen, the incident stress pulse generates both reflected and transmitted stress pulses. Strain gauges attached to the incident bar record the incident and reflected strain signals, while those attached to the transmission bar record the transmitted strain signal. These signals are collected and processed using a strain data acquisition system, as illustrated in Figure 6.

**Figure 6:**
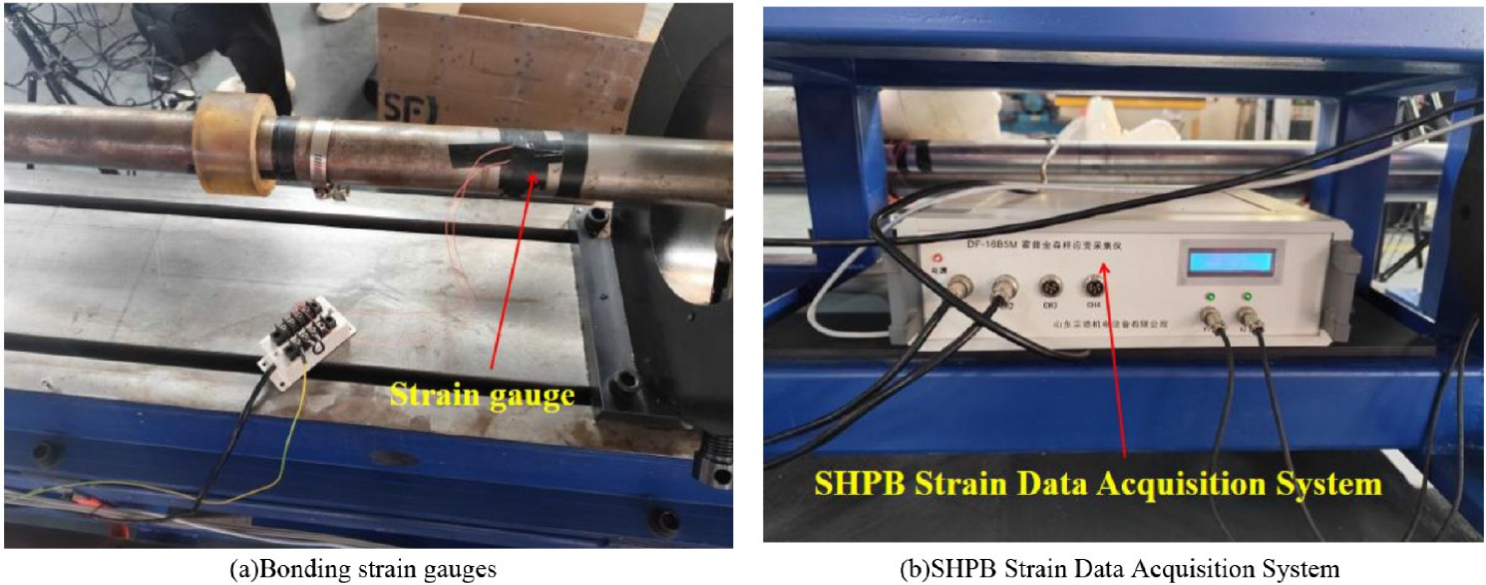
Dynamic strain acquisition system. (a) Bonding strain gauges. (b) SHPB strain data acquisition system.

Given that large-diameter SHPB systems exhibit significant dispersion effects, brass pulse shapers were employed to generate a quasi-sinusoidal loading wave, mitigating stress and deformation nonuniformity. When the incident bar is impacted, the brass deforms substantially, absorbing part of the energy and smoothing the initially steep rectangular pulse into a gradual waveform. This increase in the rise time of the incident wave ensures that the specimen achieves stress equilibrium before failure. The brass shaper is affixed at the center of the incident bar impact end using grease, effectively filtering out the high-frequency components of the rectangular wave and converting it into a smoother half-sine wave. This allows the experimental setup to provide a more consistent strain .^36–38^

Based on the above fundamental assumptions and experimental conditions, the “three-wave method” is used to process the data. By utilizing the strain signals obtained from strain gauges attached to the incident and transmission bars, the specimen’s dynamic mechanical parameters— stress *σ_s_* (*t*), strain *ε*_*s*_(*t*), and strain rate *ε̇*_*s*_(*t*)—can be indirectly calculated. The equations for the three-wave method are shown in eq 1.

1 $$\{\begin{array}{l} \sigma_{s}\left(t\right)=\frac{E_{0}A_{0}}{2A_{s}}\left[\varepsilon_{i}\left(t\right)+\varepsilon_{r}\left(t\right)+\varepsilon_{t}\left(t\right)\right]\\ \varepsilon_{s}\left(t\right)=\frac{C_{0}}{L_{s}}\int_{0}^{\tau }\left[\varepsilon_{i}\left(t\right)-\varepsilon_{r}\left(t\right)-\varepsilon_{t}\left(t\right)\right]\\ \varepsilon_{s}˙\left(t\right)=\frac{C_{0}}{L_{s}}\left[\varepsilon_{i}\left(t\right)-\varepsilon_{r}\left(t\right)-\varepsilon_{t}\left(t\right)\right] \end{array}$$

E_0_ is the elastic modulus of the pressure bars, in GPa;

*A*_0_ and *A_s_* are the cross-sectional areas of the pressure bar and the specimen, respectively, in m^2^;

*ε_i_* (*t*) is the incident strain;

*ε_r_* (*t*) is the reflected strain;

*ε_t_* (*t*) is the transmitted strain;

*C*_0_ is the wave velocity in the bar, in m/s;

*L_s_* is the initial length of the specimen, in meters.

By adjusting the gas pressure in the emission device, the impact velocity of the spindle-shaped projectile can be controlled. To ensure the reliability of the dynamic mechanical test results of conglomerate under impact loading, a Split Hopkinson Pressure Bar (SHPB) verification test was conducted prior to the actual experiments, producing a one-dimensional stress wave equilibrium curve as shown in Figure 7. The waveform illustrates that the incident and reflected waves, after superposition, closely match the transmitted wave, indicating that stress equilibrium was achieved in the SHPB system during specimen loading and thereby confirming the reliability of the dynamic test results.

**Figure 7:**
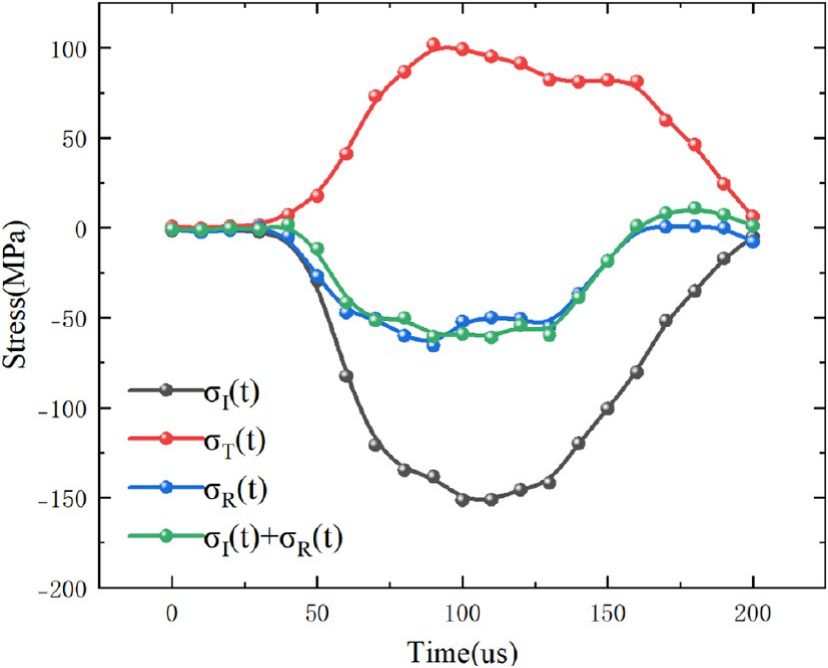
Stress balance test.

In order to ensure uniform heating of the specimens, the heating rate was set to 10 °C/min. Once the target temperature was reached, it was maintained for 1 h. Subsequently, the launch device was activated, and after the impact test was completed, the metal cage containing the conglomerate fragments was removed. After the fragments had completely cooled, all rock debris was collected, organized, and bagged with appropriate labeling.

To investigate the dynamic behavior of conglomerate under the coupled action of high temperature and impact loading, six temperature levels and six impact air pressure levels were applied in impact rock-breaking tests. The real-time temperatures were set at 25 °C, 100 °C, 150 °C, 200 °C, 250 °C, and 300 °C, while the impact air pressures were 0.12, 0.15, 0.18, 0.20, 0.22, and 0.23 MPa. Each variable was tested three times.

## 3 Experimental Results and Discussion

The incident, transmitted, and reflected wave signals collected were processed to obtain the stress–strain curves of the conglomerate at different temperatures. The corresponding peak strain (*ε_p_*), strain rate (*ε̇_s_*), compressive strength (*σ_d_*), and elastic modulus (E) can all be obtained from the stress–strain curves.

### 3.1 Effect of Temperature on Dynamic Mechanical Parameters

Figure 8 shows that the stress–strain curves of conglomerate under different temperatures (25–300 °C) and strain rates exhibit the typical pattern of initial elastic deformation, followed by plastic deformation and eventual postpeak softening, with stress rising to a peak and then gradually decreasing as strain develops. Temperature exerts a pronounced weakening effect on rock strength: at relatively low temperatures (25–150 °C), the peak stress remains high, indicating that the rock structure is still relatively intact and thermal damage is limited; when the temperature increases to 200–250 °C, the peak stress drops markedly, suggesting that thermal expansion induces microcrack initiation and propagation along mineral interfaces, thereby reducing the load-bearing capacity. Notably, at 200 °C, the postpeak decline is slower than at other temperatures. This phenomenon may be attributed to moderate thermal damage: microcracks have begun to form but have not yet fully coalesced, allowing gradual stress release rather than abrupt failure. Differential thermal expansion among mineral components may also induce local compressive stresses, and part of the energy may be dissipated through microcrack closure or localized plastic deformation, further moderating the postpeak stress decline. At 300 °C, the peak stress reaches its minimum, and the overall curve shifts downward, reflecting severe structural degradation and strength deterioration caused by high-temperature damage. By contrast, strain rate has a clear strengthening effect under all temperature conditions: at the same temperature, higher strain rates lead to higher peak stresses, demonstrating a typical strain-rate hardening behavior. Even at elevated temperatures (e.g., 300 °C), increasing the strain rate can still significantly enhance peak stress, although it cannot fully counteract the strength reduction caused by thermal damage. Overall, the dynamic response of conglomerate under high-temperature impact loading is governed by the coupled effect of thermal softening and strain-rate hardening, whereby elevated temperatures markedly reduce strength, while higher strain rates partially mitigate this reduction, jointly controlling its dynamic mechanical behavior.

**Figure 8:**
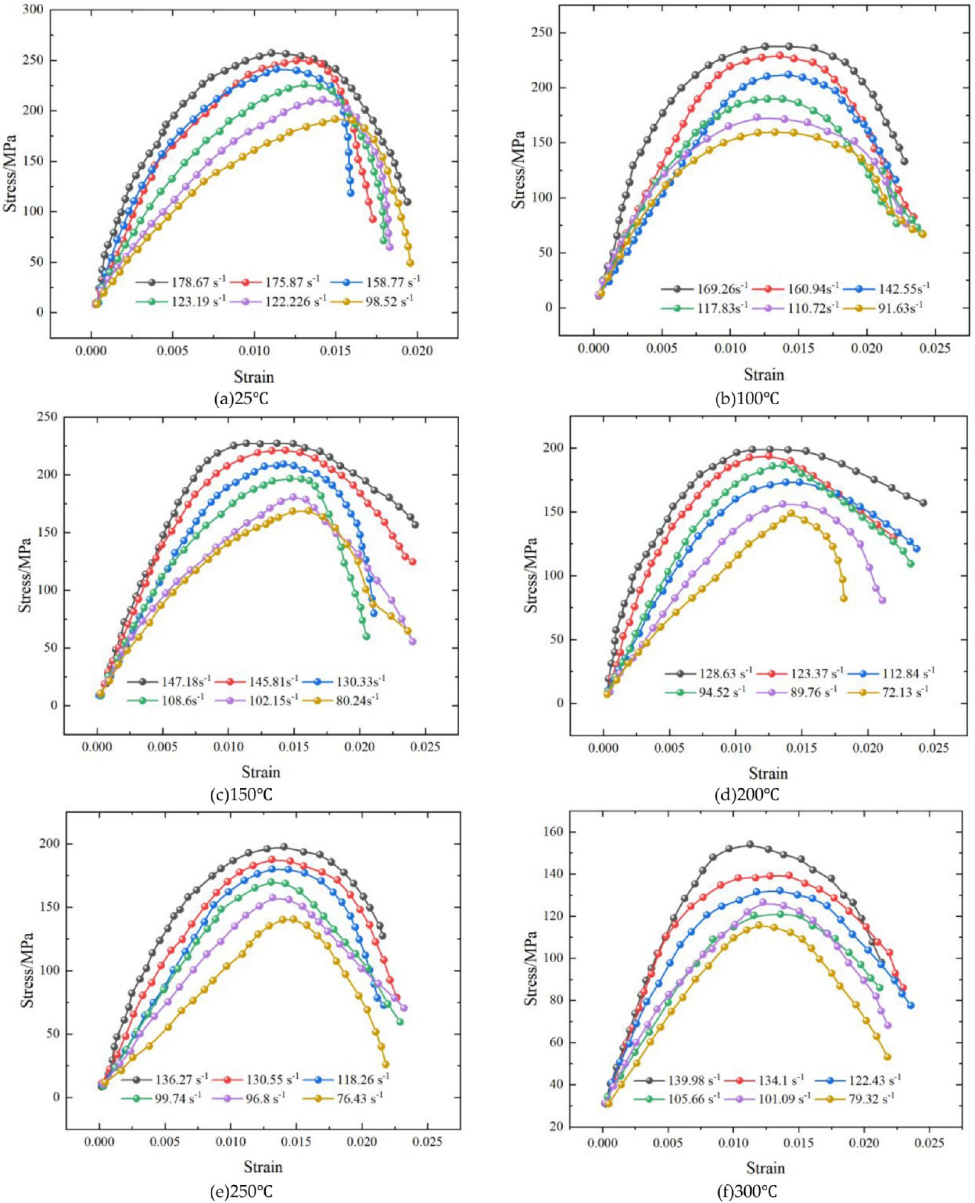
Stress–strain curves of conglomerate at different temperatures and strain rates. (a) 25 °C; (b) 100 °C; (c) 150 °C; (d) 200 °C; (e)­250 °C; (f) 300 °C.

### 3.2 Effect of Temperature on Energy Dissipation Characteristics

During the impact process of the Split Hopkinson Pressure Bar (SHPB) test, both the incident and transmission bars are made of steel, which largely remains within the elastic deformation range during the experiment. Therefore, it can be reasonably assumed that there is negligible energy loss between the measurement points on the incident and transmission bars and their respective impact interfaces. As a result, the total energy generated during the test can be divided into three parts: the energy of the incident wave, the energy of the transmitted wave, and the energy absorbed by the rock specimen. This absorbed energy is the primary contributor to the fragmentation and failure of the specimen. It is this portion of dissipated energy that causes irreversible damage to the rock and cannot be recovered or released. The incident energy *W_I_*, reflected energy *W_R_*, transmitted energy *W_T_*, and absorbed energy *W_A_* of the conglomerate specimen can be calculated by integrating the strain of the incident, reflected, and transmitted waves measured on the pressure bars.^39–42^

2 $$\{\begin{array}{l} W_{I}=\frac{A_{b}C_{0}}{E_{b}}\int_{0}^{t}\sigma_{I}^{2}dt=AE_{0}C_{0}\int_{0}^{t}\varepsilon_{I}^{2}dt\\ W_{R}=\frac{A_{b}C_{0}}{E_{0}}\int_{0}^{t}\sigma_{R}^{2}dt=AE_{0}C_{0}\int_{0}^{t}\varepsilon_{R}^{2}dt\\ W_{T}=\frac{A_{b}C_{0}}{E_{0}}\int_{0}^{t}\sigma_{T}^{2}dt=AE_{0}C_{0}\int_{0}^{t}\varepsilon_{T}^{2}dt \end{array}$$

where t is time (s); A_b_is the cross-sectional area of the pressure bars (m^2^);C_0_ is the wave propagation velocity in the bars (m/s);C_b_ is the wave propagation velocity in the bars (m/s);*E*_0_ is the elastic modulus of the bars (GPa); *σ_I_*, *σ_R_* and *σ_T_* are the incident, reflected, and transmitted stresses (MPa), respectively; *ε_I_*, *ε_R_*, and *ε_T_* are the corresponding strains of the incident, reflected, and transmitted waves in the bars.

According to the law of energy conservation, the dissipated energy can be calculated using eq 3, and the corresponding energy dissipation density is given by eq 4.

3 $$W_{S}=W_{I}-W_{R}-W_{I}$$

4 $$\rho_{W}=\frac{W_{S}}{V_{S}}$$

where *V_S_* is the volume of the conglomerate specimen.

Figure 9 presents the dynamic stress–strain responses of conglomerate under varying temperature and impact air pressure conditions. The results demonstrate that both parameters exert significant influences on the mechanical behavior, and a distinct coupling effect can be observed.

**Figure 9:**
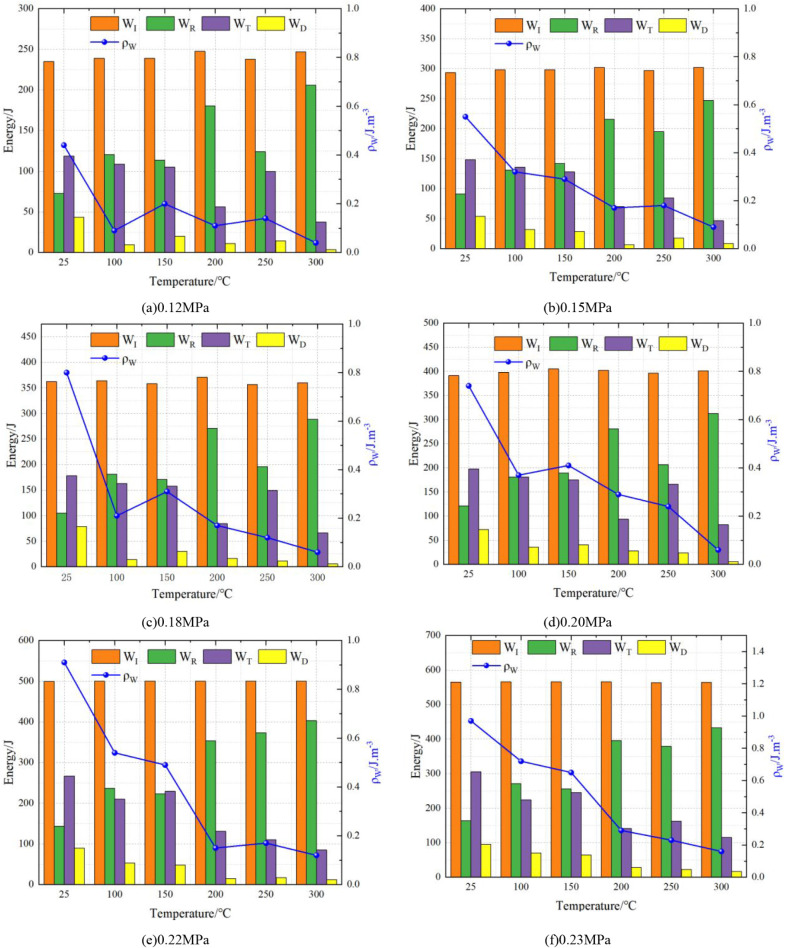
Relationship between temperature and energy variation in conglomerate under different impact air pressures: (a) 0.12 MPa, (b) 0.15 MPa, (c) 0.18 MPa, (d) 0.20 MPa, (e) 0.22 MPa, and (f) 0.23 MPa.

#### 3.2.1 Effect of Impact Air Pressure

With increasing impact pressure, the dynamic strength of conglomerate gradually rises. At low temperatures (25–150 °C), this enhancement exhibits an approximately linear trend, indicating that higher loading rates promote brittle fracture. However, once the temperature exceeds 200 °C, the strengthening effect of pressure tends to diminish, suggesting that elevated temperature alters the failure mode and suppresses stress transfer efficiency. This observation is consistent with previous findings that high-temperature conditions reduce rock brittleness and limit dynamic strength development.^43^

#### 3.2.2 Effect of Temperature

Temperature exerts a 2-fold influence. As it rises from 25 to 150 °C, conglomerate exhibits more pronounced brittleness, and microcracks are more easily initiated and propagated, thereby lowering impact resistance and enhancing fragmentation efficiency. In contrast, at 200–300 °C, thermal decomposition or softening of cementing materials leads to increased plasticity, greater energy dissipation, and reduced rock-breaking efficiency. This brittle-to-ductile transition at elevated temperatures aligns with thermal damage mechanisms reported for sandstones and granites.^44^

#### 3.2.3 Coupling Effects

The combined influence of temperature and pressure reveals nonlinear dynamic behavior. Under low-temperature and high-pressure conditions, conglomerate undergoes efficient fragmentation with typical brittle failure. Conversely, under high-temperature and low-pressure conditions, the dynamic response is significantly weakened. At high temperatures combined with high pressures, the curves display a “plateau” or “inflection point,” indicating that the beneficial effect of pressure is counteracted by thermal weakening. Such nonlinear interactions highlight the complexity of rock behavior in deep, hot formations and are of practical significance for drilling engineering.

In summary, the dynamic mechanical response of conglomerate is strongly dependent on both thermal and loading conditions. Low temperatures facilitate brittle fragmentation under impact loading, while high temperatures enhance energy dissipation and suppress effective breakage. These findings not only enrich the understanding of thermomechanical coupling in conglomerates but also provide guidance for the optimization of drilling and rock-breaking strategies in deep, high-temperature reservoirs.

## 4 Numerical Simulation and Discussion

### 4.1 Conglomerate SHPB Model Establishment

Assuming that the pebbles in the conglomerate are spherical, in order to meet the grading requirements of pebbles of different sizes in the conglomerate, the Fuller grading curve is typically used to determine the grading relationship of the pebbles in the mesoscale model of the conglomerate, as shown in eq 5.^45^

5 $$P\left(d\right)=100{\left(d/d_{\max}\right)}^{k}$$

Where *P*(*d*) represents the cumulative percentage of pebbles with a diameter less than d(%); *d*_max_ is the maximum aggregate diameter (mm); and k is the exponent constant, where 0.45<*k*<0.7.

To obtain the maximum density ideal grading of the pebbles, the grading curve for n = 0.5 is selected to determine the content of pebbles in the conglomerate, as shown in Figure 10.^46,47^

**Figure 10:**
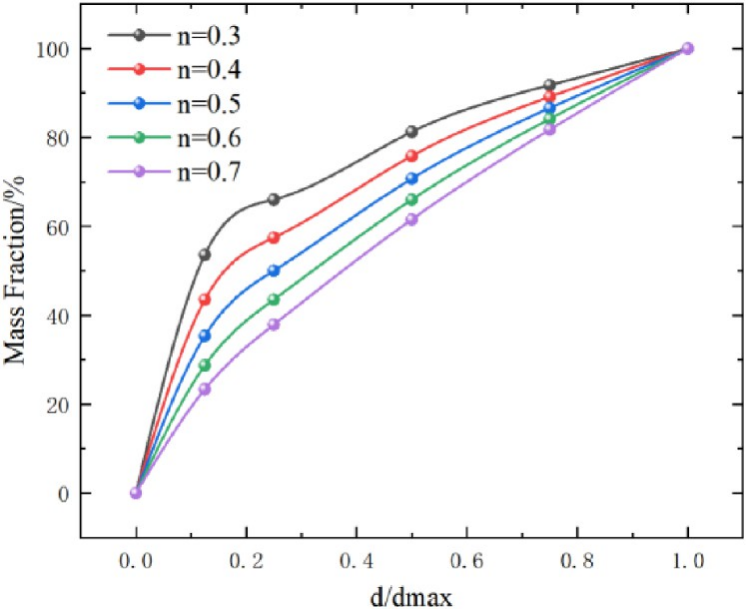
Fuller grading curves.

Based on the pebble grading curve, the number of pebbles of different diameters within the selected size range is determined. The pebbles are then placed in the specified area in descending order of particle size. The calculation method for the number of pebbles of different sizes is shown in eq 6.

6 $$P\left[d_{s},d_{s+1}\right]=\frac{P\left(d_{s+1}\right)-\left(d_{s}\right)}{P\left(d_{\max}\right)-\left(d_{\min}\right)}$$

Where *P*[*d_s_*,*d_s_*_+1_] is the content of pebbles with a particle size between [*d_s_*,*d_s_*_+1_], *P*(*d*_max_) is the content of pebbles with the maximum particle size, and *d*_min_ is the content of pebbles with the minimum particle size.

In order to determine the content of pebbles in the conglomerate sample, the center profile of the conglomerate is used as the reference. The conglomerate is divided into 8 equal parts along the circumference, and markings are made accordingly. A side view of the sample is then captured using a camera, and the side view is stitched together according to the marked points, ultimately obtaining the unfolded side view of the conglomerate sample. The unfolded side view is imported into ImageJ software, and after adjusting the grayscale values, the pebble contours are obtained, as shown in Figure 11. As can be seen from the figure, the contour of the blank area in the grayscale image closely matches the contour in the unfolded actual image. By calculating the area of the blank region, the percentage of pebbles in the sample relative to the total conglomerate is found to be 26.709%. Based on the grading relationship and using eqs 8 and 9, it is calculated that the pebbles with particle sizes of 2–6 mm, 6–10 mm, 10–20 mm, and 20–30 mm account for 6.92%, 10.57%, 19.73%, and 15.57% of the total mass of the conglomerate, respectively.

**Figure 11:**
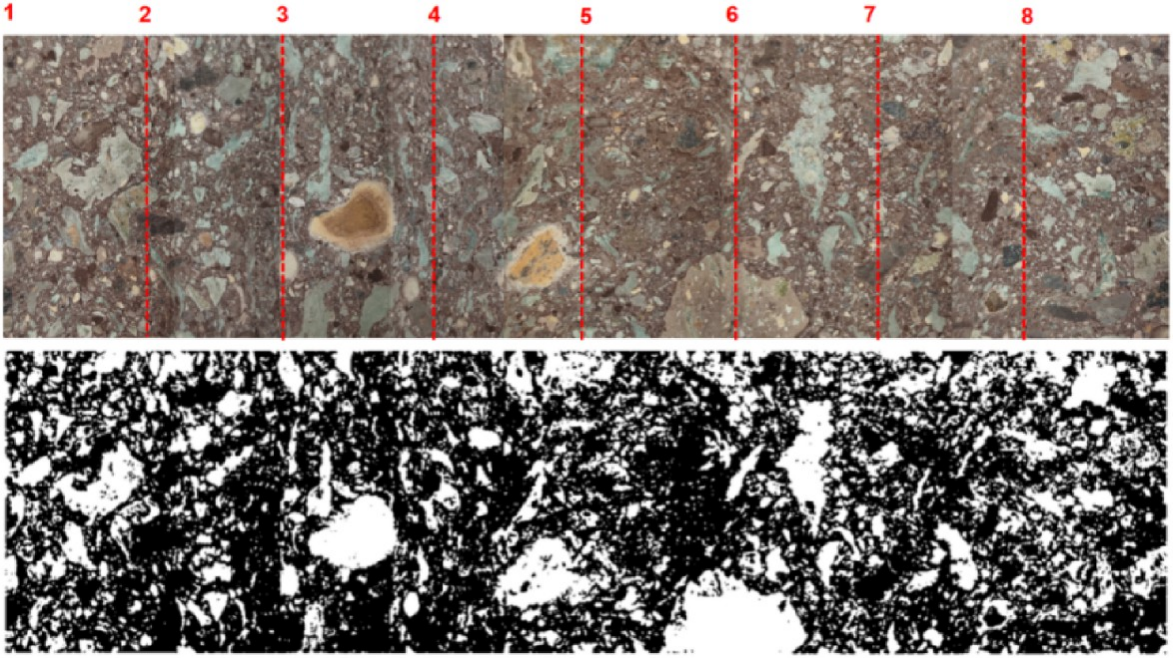
Unfolded side view of the conglomerate sample.

After determining the cumulative percentage of pebbles within each particle size grading range, a Fortran script program is used to randomly place the pebbles within the predefined region *x*_min_ ∈[*x*_min_, *x*_max_], *y*_min_ ∈[*y*_min_, *y*_max_], and *z*_min_ ∈[*z*_min_, *z*_max_]. During the placement process of the pebbles, two conditions must be met simultaneously: ①there is no overlap between the pebbles; ② the pebbles do not intersect with the boundary of the container. These conditions are controlled by formula (7).

7 $$\{\begin{array}{l} \sqrt{{\left(x_{m}-x_{n}\right)}^{2}+{\left(y_{m}-y_{n}\right)}^{2}+{\left(z_{m}-z_{n}\right)}^{2}}>\left(\frac{d_{m}+d_{n}}{2}\right)\\ x_{m}=\left(r-r_{m}\right)\times n_{1}\times \cos\left(2\pi \times n_{2}\right)\\ y_{m}=\left(r-r_{m}\right)\times n_{1}\times \cos\left(2\pi \times n_{2}\right)\\ z_{m}=z_{D}+r_{m}+\left(Z_{T}-Z_{D}-2\times r_{m}\right)\times n_{3} \end{array}$$

Where *x_m_*, *y_m_*, *z_m_* are the coordinates of the m-th pebble particle; *d_m_* is the diameter of the m-th pebble particle (mm); *r* is the radius of the cylindrical conglomerate specimen, which is taken as 50 mm in this study; *r_m_* is the radius of the m-th pebble particle (mm); *z_D_* and *z_T_* represent the coordinates of the bottom and top surfaces of the cylindrical specimen, respectively; and *n*_1_, *n*_2_, *n*_3_ are random numbers between 0 and 1.

The numerical model of conglomerate constructed using the three-dimensional mapped mesh algorithm is shown in Figure 12. The generated model contains 433,956 hexahedral elements and 449,396 nodes. Under the condition of 32 CPUs, the dynamic impact simulation required 36 min of computation time. Therefore, meshing the model with hexahedral elements significantly reduces the number of elements and nodes, saving computational resources and greatly improving simulation efficiency.

**Figure 12:**
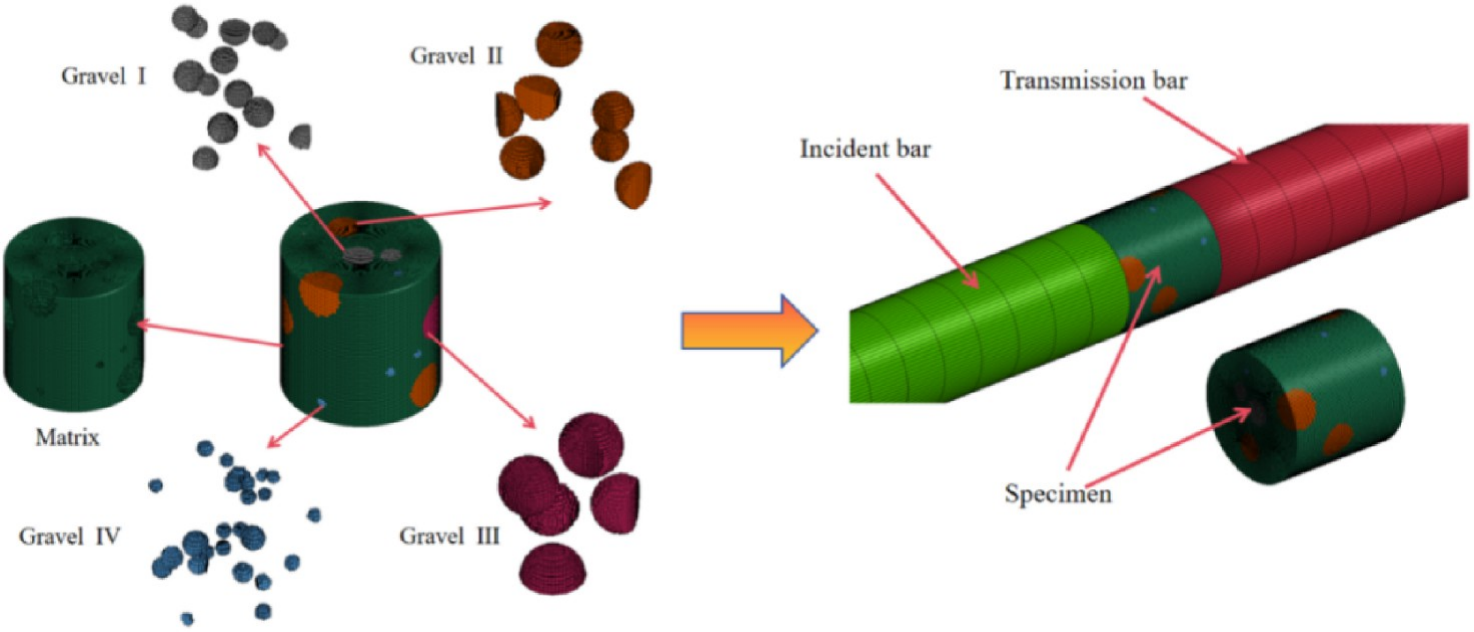
Numerical model of gravel rock.

To simulate the impact loading, a half-sine wave was directly applied at the incident end of the incident bar instead of modeling a spindle-shaped projectile. The contact between the specimen and the bars was defined using the *CONTACT_ERODING_SURFACE_ TO_SURFACE_ID keyword, which automatically handles surface-to-surface contact. The failure of the conglomerate specimen was represented by element deletion through the *MAT_ADD_EROSION keyword.

Temperature induces irreversible changes in the mechanical properties of conglomerate; hence, the effect of temperature must be considered when simulating SHPB tests under different thermal conditions. In this study, the “implicit–explicit sequential solution” approach was used to simulate the impact failure of conglomerate under real-time temperature conditions. As is well-known, the ANSYS implicit method is efficient for solving static problems, whereas transient problems require explicit methods. The “implicit–explicit sequential solution” essentially involves writing the results of the implicit solution into an ASCII drelax file. ANSYS/LS-DYNA then reads this deformation data to initialize the geometric model, after which transient dynamic analysis is performed.^47^ The specific calculation process is illustrated in Figure 13.

**Figure 13:**
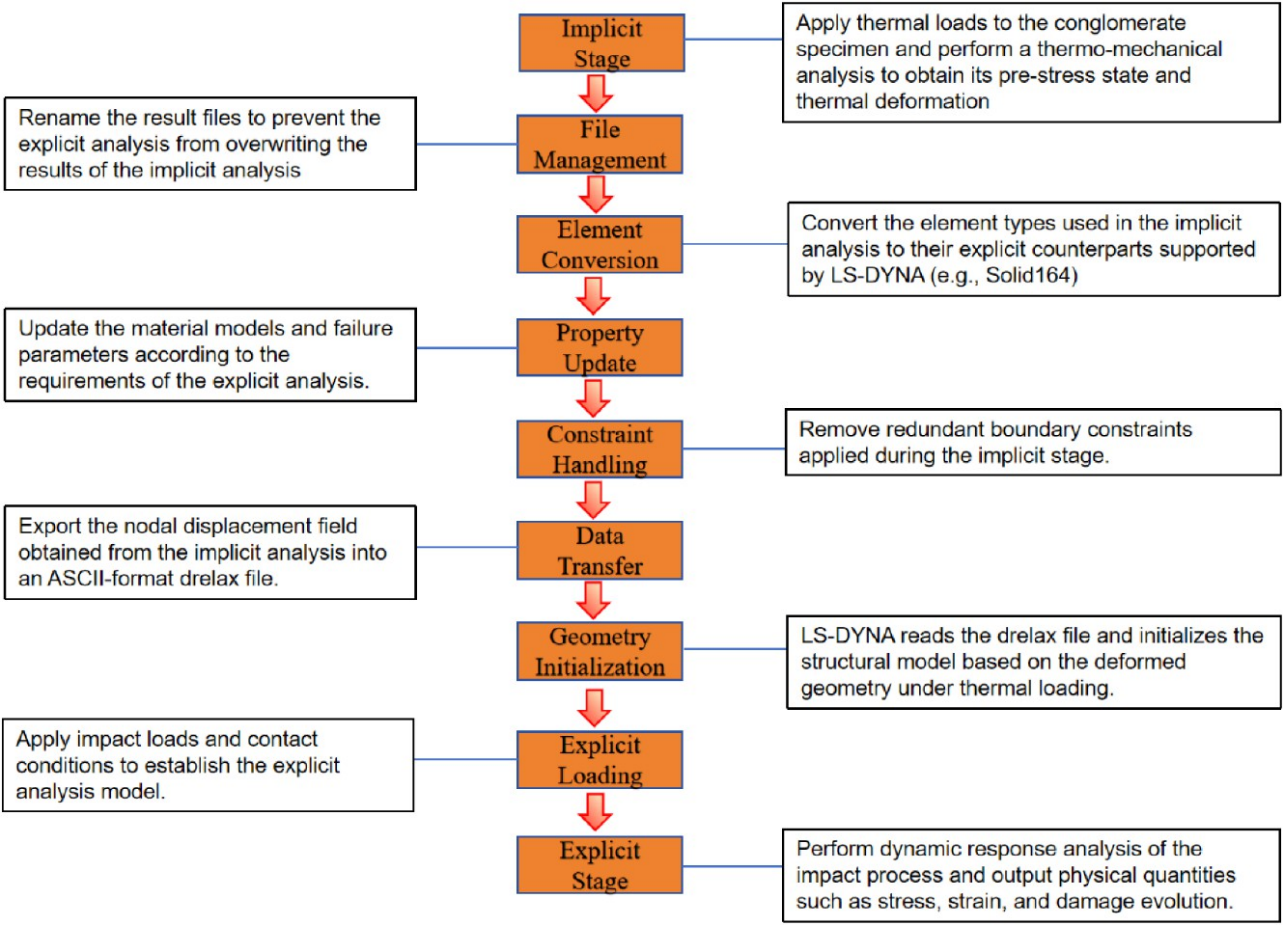
Implicit–explicit sequential solving process.

### 4.2 Determination of HJC Model Parameters for Conglomerate

The Holmquist–Johnson–Cook (HJC) model consists of three main components: a strength model, a damage model, and an equation of state.^48^ Due to its ability to effectively simulate the nonlinear response of rock-like materials under impact loading, it has been widely used in related studies, as illustrated in Figure 14.

**Figure 14:**
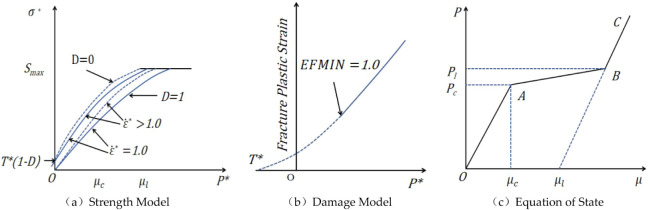
HJC model of conglomerate: (a) strength model; (b) damage model; (c) equation of state.

#### 4.2.1 Strength Model

The strength model of the HJC (Holmquist–Johnson–Cook) model describes the normalized equivalent stress, and its mathematical expression is given by.

8 $$\sigma^{*}=\left[A\left(1-D\right)+{\mathrm{BP}}^{*N}\right]\left[1+\mathrm{Cln}\left({\mathrm{\varepsilon ̇}}^{*}\right)\right]\leq S_{\max}$$

In this equation, *σ* ^*^ and *P* ^*^ represent the normalized equivalent stress and normalized pressure, respectively. Specifically, *σ* ^*^ = *σ*/*f_c_*, where *σ* is the actual equivalent stress, and *P* ^*^ = *p*/*f_c_*, where *p* is the hydrostatic pressure. *f_c_* denotes the quasi-static uniaxial compressive strength of the material. $\varepsilon^{*}˙$ is the normalized strain rate, defined as $\varepsilon^{*}˙=\mathrm{\varepsilon ̣}/{\mathrm{\varepsilon ̣}}_{0}$ ; ε̣ is the applied strain rate, and ${\mathrm{\varepsilon ̣}}_{0}$ is the reference strain rate. D is the damage parameter, which is determined by the damage evolution equation. A, B, C, and N are material strength parameters representing the normalized cohesive strength, pressure hardening coefficient, strain rate sensitivity exponent, and pressure hardening exponent, respectively. *S*_max_ is the maximum normalized equivalent stress.

#### 4.2.2 Damage Model

In the HJC model, material damage accumulates through plastic strain, which includes both shear plastic strain (i.e., equivalent plastic strain) and volumetric plastic strain, as illustrated in Figure 14b. The damage evolution is defined by eq 9:

9 $$D=\sum \left(\Delta \varepsilon_{P}+\Delta \mu_{P}\right)/\left(\varepsilon_{P}^{f}+\mu_{P}^{f}\right)$$

where Δ*_εP_* and Δ*_μP_* represent the equivalent plastic strain increment and volumetric plastic strain increment in a given computational cycle, respectively. $\varepsilon_{P}^{f}$ and $\mu_{P}^{f}$ denote the equivalent plastic strain and volumetric plastic strain at failure under ambient pressure conditions.

#### 4.2.3 Equation of State

The equation of state (EOS) in the HJC model is typically represented in a piecewise form, as shown in Figure 14c, and consists of three distinct stages:

Stage I (Segment OA): This is the linear elastic stage, during which the material obeys Hooke’s law, and no irreversible deformation occurs.

Stage II (Segment AB): This is the compaction transition stage, where the initial voids within the material begin to close, and plastic deformation initiates.

Stage III (Segment BC): This is the fully compacted stage, characterized by the near-complete closure of internal pores and a highly nonlinear response. In this regime, the relationship between hydrostatic pressure and volumetric strain is typically described using a third-order polynomial, as given in eq 10.

10 $$P=K_{1}\mu +K_{2}\mu^{2}+K_{3}\mu^{3}$$

Here, *P_c_* and *μ_c_* represent the hydrostatic pressure and volumetric strain corresponding to pore collapse, while *P_l_* and *μ_l_* denote the hydrostatic pressure and volumetric strain at the compaction limit of the rock. *K*_1_, *K*_2_ and *K*_3_ are material constants associated with pressure.

However, the HJC model involves up to 21 parameters, making the determination process relatively complex. Common methods for parameter identification include: ① Direct measurement of certain parameters through physical and mechanical testing of rock materials, such as tensile and compressive tests; ② ② Referring to the original HJC model parameters and modifying them based on specific engineering experience; ③ ③ Repeated numerical simulations for parameters with high sensitivity to iteratively calibrate the model results against experimental data (i.e., inverse determination).^42^ Among these, Method ① requires extensive laboratory work and is often time-consuming and difficult to implement. Therefore, this study adopts a hybrid approach combining Methods ① and ③ to improve the efficiency and accuracy of parameter identification.

Due to the lack of triaxial compression test data for conglomerate at elevated temperatures (100–300 °C), parameters A, B, and N could not be directly determined. Accordingly, their influence on the stress–strain response was analyzed based on room-temperature values, and multiple fitting simulations of SHPB tests were conducted using a nonlinear finite element solver. Through a “trial-and-error” iterative approach, the numerical results were closely aligned with the experimental data. Other parameters were obtained in the same manner as at room temperature. The final HJC parameters for each temperature are listed in Tables 2 and 3.

**Table 2:** Main HJC Model Parameters for Matrix Material at Different Temperatures

T_emp_(°C)	*ρ0* (*g·m* ^*‑3*^)	*G* (G*Pa*)	*f*_*c*_(*MPa*)	*T*_*1*_(*MPa*)	*P*_*crush*_ (*MPa*)	*μ*_*crush*_	*P*_*lock*_(GPa)	*μ*_*lock*_
20	2.2	10.4	50	9.6	25	0.0038	0.75	0.031
100	2.2	9.86	45	8.2	23	0.004	0.7	0.033
150	2.2	9.52	40	7.5	21	0.0043	0.65	0.034
200	2.2	9.18	35	6.3	19	0.0045	0.6	0.036
250	2.2	8.84	30	5.4	17	0.0048	0.55	0.039
300	2.2	8.5	25	4.1	15	0.0052	0.5	0.047

**Table 3:** Main HJC Model Parameters of Gravel at Different Temperatures

T_emp_(°C)	*ρ0* (*g·m* ^*‑3*^)	*G* (G*Pa*)	*f*_*c*_(*MPa*)	*T*_1_ (*MPa*)	*P*_*crush*_(*MPa*)	*μ*_*crush*_	*P*_*lock*_(GPa)	*μ*_*lock*_
20	2.68	26.6	136	10	46	0.0014	1.2	0.017
100	2.68	24.5	112	9.28	41	0.0016	1.1	0.024
150	2.68	22.5	95	8.84	38	0.0017	1.06	0.028
200	2.68	20	86	8.4	35	0.0018	1	0.031
250	2.68	17.5	80	8	32	0.0019	0.95	0.034
300	2.68	15	72	7.5	30	0.0023	0.9	0.038

### 4.3 Numerical Simulation Results and Damage Evolution

Figure 15 shows the stress–strain curves of conglomerate obtained from experiments and numerical simulations at different temperatures. It can be seen from the figure that the experimental and simulated curves are generally consistent, indicating that the numerical simulations can accurately capture the mechanical behavior of the conglomerate under varying temperature conditions. This demonstrates that the HJC model effectively represents the prefailure mechanical behavior of the conglomerate, providing a reliable basis for further investigation of its thermo–mechanical coupled response. However, the peak stress and ultimate strain obtained from the simulations still show some discrepancies compared with the experimental measurements. This is primarily due to the intrinsic heterogeneity and strong anisotropy of the conglomerate, which consists of clasts of various sizes embedded in a matrix and is further influenced by natural microdefects and pore structures, leading to local mechanical responses that differ from those predicted by the idealized model.

**Figure 15:**
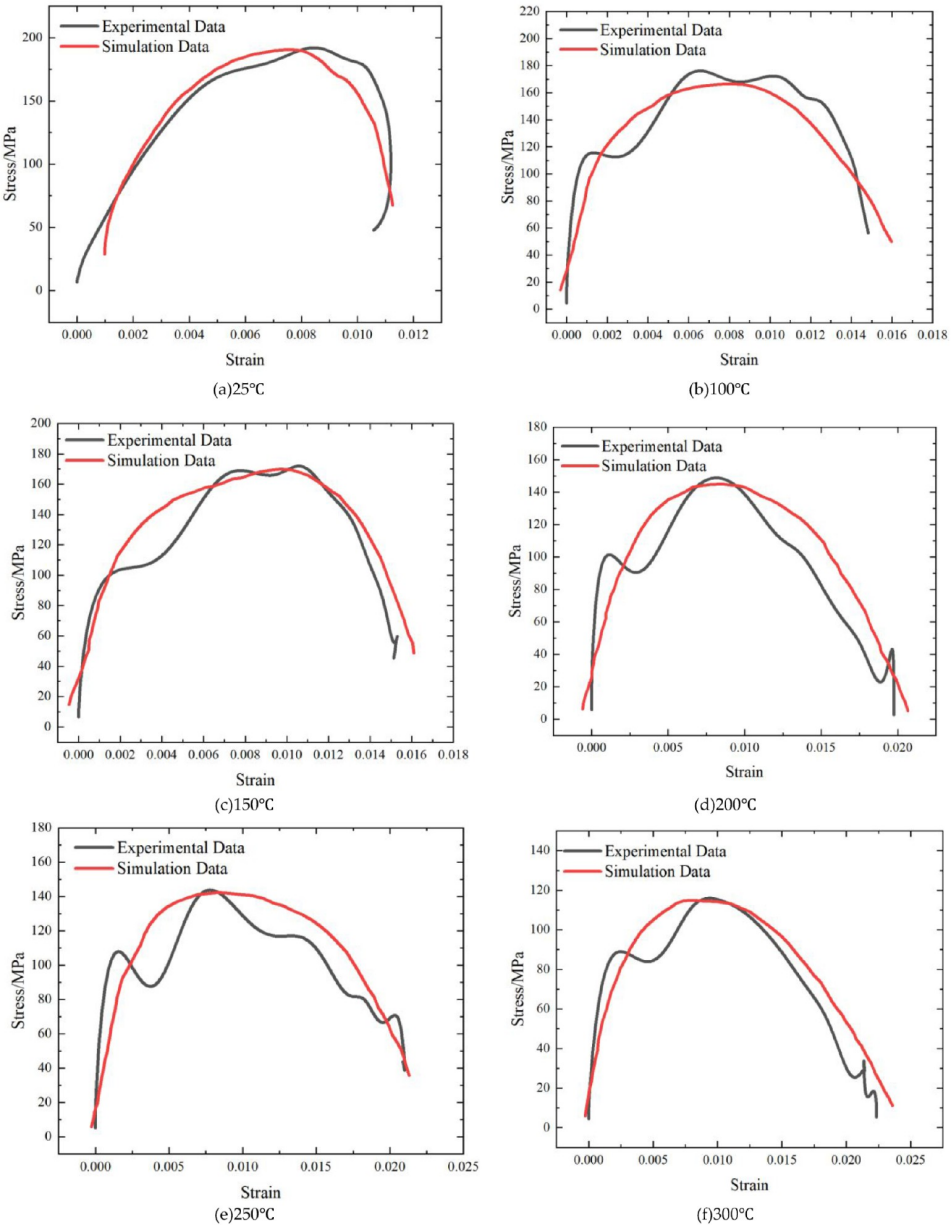
Simulation and comparison of stress–strain of samples under different temperature conditions. (a) 25 °C; (b) 100 °C; (c) 150 °C; (d) 200 °C; (e) 250 °C; (f) 300 °C.

In uniaxial dynamic compression tests conducted under different temperature conditions, the dynamic compressive strength of the rock is generally higher than its static compressive strength at the corresponding temperature. The dynamic increase factor (DIF) can be introduced to characterize the strength variation of the rock under the combined effects of temperature and dynamic loading, as expressed in eq 11:

11 $$\mathrm{DIF}=\frac{\sigma_{d}}{\sigma_{s}}$$

where *σ_d_* is the dynamic compressive strength and *σ_s_* is the static compressive strength.

Figure 16 illustrates the variation of DIF for conglomerate specimens under different temperature conditions. As shown in the figure, the numerically simulated DIF values exhibit an overall trend consistent with the experimental results, both demonstrating a gradual decrease in DIF with increasing temperature. This indicates good agreement between the two in capturing the influence of temperature on dynamic strength. The slight discrepancies observed under a few conditions may be attributed to simplified material parameters, the intrinsic heterogeneity of the specimens, and the challenges of fully representing high-temperature microcrack evolution within the numerical model.

**Figure 16:**
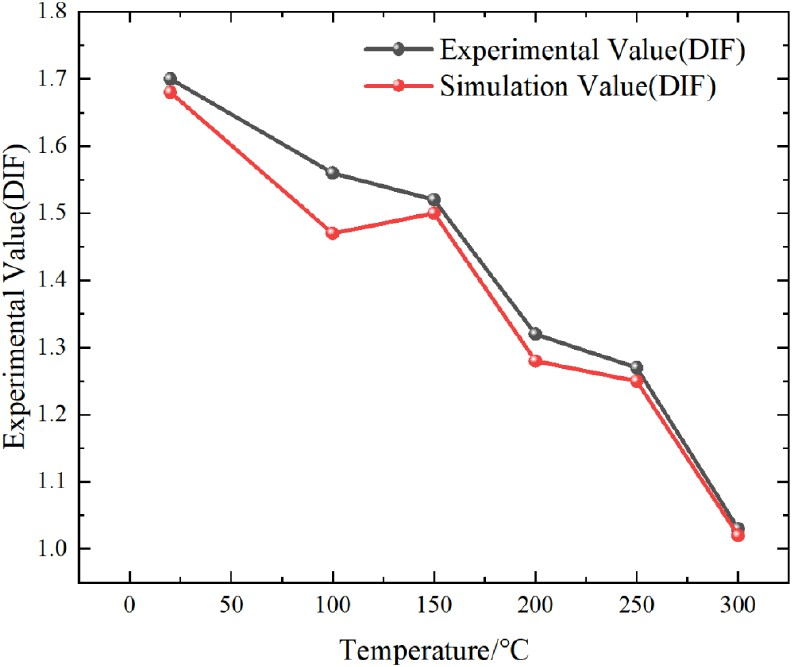
Comparison between the experimental and simulated DIF values of the specimens.

Figure 17 presents the stress evolution cloud maps of conglomerate specimens under different temperatures (25 °C, 100 °C, 150 °C, 200 °C, 250 °C, and 300 °C) and at various time steps, as well as the fractal fragments obtained from the specimens at different temperatures. In the stress cloud maps, red indicates maximum stress, while blue represents minimum stress. Taking the specimen at 25 °C as an example, at 750 μs, no obvious macroscopic failure has occurred; however, slight stress concentrations can be observed around the clasts, particularly at the clast–matrix interfaces and local bottom regions, as indicated by the red–orange areas in the Z-stress cloud map. The maximum compressive stress is 73.13 MPa, and the maximum tensile stress is 2.326 MPa. At this stage, stress waves begin to propagate within the clasts and along their interfaces, while the matrix remains compacted with no visible macroscopic cracks. By 850 μs, cracks initiate at the clast–matrix interfaces, especially vertically propagating through the matrix between clasts. At this time, the maximum compressive stress is 27.2 MPa, and the maximum tensile stress is 56.8 MPa. Shear bands on the specimen surface become prominent, partial detachment of clasts occurs, and damage and cracking also appear along the bottom and side boundaries, indicating evident tensile and shear failure. At 850 μs, cracks rapidly propagate, forming multiple axially aligned failure zones. The matrix experiences extensive fragmentation, with block detachment and edge spalling observed at clast boundaries, some of which are fully separated. A primary fracture zone has formed at this stage, and the energy concentration regions (shown by the bands from green to light blue) indicate partial stress release. By 1000 μs, the axial penetration failure band is fully developed, the matrix is extensively fragmented, and clast edge spalling and interface debonding confirm that the primary failure structure has been completely established.

**Figure 17:**
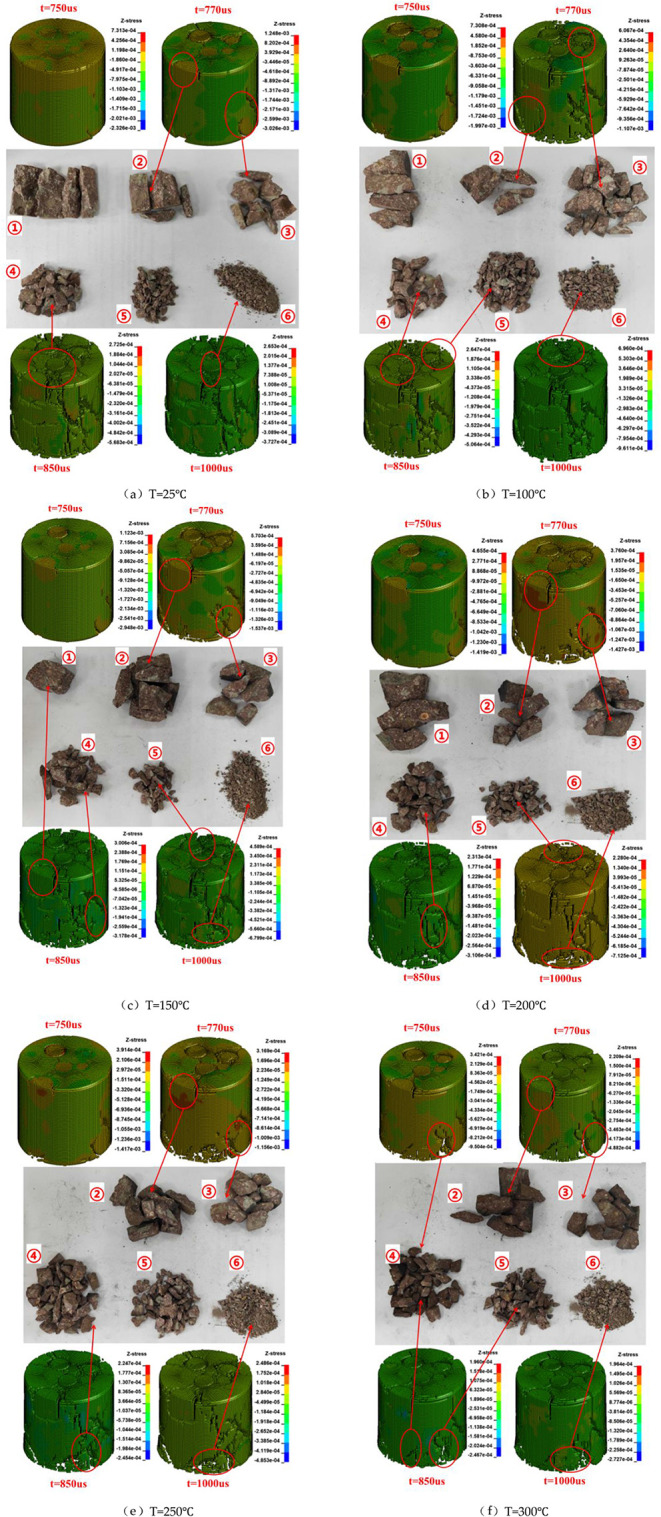
Stress evolution cloud maps and fractal fragments of conglomerate specimens under different temperatures and at various time steps. (a) 25 °C; (b) 100 °C; (c) 150 °C; (d) 200 °C; (e) 250 °C; (f) 300 °C.

Comparing the stress cloud maps at the same time step (t = 770 μs) under different temperatures reveals a clear temperature-dependent impact response. As temperature increases, interface damage between clasts and the matrix becomes more pronounced, evolving from minor shear cracks to through-thickness delamination. Crack propagation and block fragmentation within the matrix are also intensified. The stress distribution maps indicate that at room temperature, stress release is limited and stress concentration is localized, whereas at higher temperatures, stress rapidly diffuses and becomes more uniform, suggesting a significant reduction in material stiffness and a shift in energy dissipation from elastic deformation to plastic crushing. In particular, at temperatures of 250 °C and above, the failure mode exhibits characteristics of “free fragmentation,” indicating a complete loss of load-bearing capacity of the conglomerate structure.

The fragments resulting from the conglomerate failure were classified into size ranges of ① 40–50 mm, ② 30–40 mm, ③ 20–30 mm, ④10–20 mm, ⑤5–10 mm, and ⑥ 0–5 mm. Comparing the fractal fragments at different temperatures shows that higher temperatures produce a greater proportion of smaller fragments, indicating that the increase in temperature directly exacerbates the degree of specimen fragmentation. These observations are generally consistent with the results obtained from numerical simulations, further validating the accuracy of the HJC-based modeling approach.

Overall, the combined experimental and numerical results demonstrate that temperature has a critical influence on the impact-induced failure mechanisms of conglomerates. With increasing temperature, not only does the interface damage between clasts and matrix intensify, but the matrix itself also undergoes more extensive cracking and fragmentation. This leads to accelerated energy dissipation through plastic deformation and particle crushing, ultimately resulting in a transition from localized damage at lower temperatures to widespread “free fragmentation” at elevated temperatures. Such insights are essential for understanding the thermomechanical behavior of heterogeneous rock materials under dynamic loading conditions and provide a reliable basis for predicting their failure patterns in engineering applications.

## 5 Conclusions

This study employed a ⌀50 mm split Hopkinson pressure bar (SHPB) system to conduct systematic dynamic tests on conglomerate specimens under different temperatures (25 °C–300 °C) and varying impact loads. Combining the experimental results with finite element numerical simulations and fractal analysis, the mechanical response and fragmentation behavior of the conglomerate under high-temperature impact loading were investigated. The main conclusions are summarized as follows:

1. Temperature-induced weakening of dynamic mechanical properties: Increasing temperature has a significant weakening effect on the dynamic compressive strength and elastic modulus of the conglomerate. In the range of 25 to 150 °C, thermal damage is relatively limited, and the rock structure remains largely intact. When the temperature rises above 200 °C, differential thermal expansion among minerals leads to microcrack propagation at the clast–matrix interfaces, resulting in a noticeable reduction in load-bearing capacity. At 300 °C, strength degradation is most severe, as indicated by the overall downward shift of the stress–strain curves.
2. Transition in energy evolution mechanisms: Under low-temperature conditions (≤150 °C), the conglomerate exhibits higher energy absorption and dissipation capacity, facilitating crack propagation and effective energy release. In contrast, under high-temperature conditions (≥200 °C), energy reflection is enhanced while dissipation decreases, leading to more brittle failure and reduced energy utilization efficiency. This indicates the existence of a critical temperature transition point (approximately 150 °C) that governs the shift of the energy response mechanism from “plastic dissipation” to “brittle reflection.”
3. Numerical reproduction of mesoscale failure processes: A mesoscale numerical model of the conglomerate, based on Fuller grading and the HJC model, successfully reproduced the dynamic stress–strain response under different temperatures. The simulation clearly revealed that failure initiates at stress concentrations around the clast–matrix interfaces, followed by crack propagation, coalescence, and the formation of axial principal fracture bands. With increasing temperature, interface damage intensifies and matrix fragmentation becomes more extensive, resulting in a transition of the failure mode from localized damage to “overall fragmentation.” This highlights temperature as a key factor controlling the degree of impact-induced failure in the conglomerate.

In summary, this study provides a deeper understanding of the mechanical response characteristics of conglomerate under coupled high-temperature and impact loading, which is of significant importance for assessing the stability of rock layers in geothermal resource development. Accurate knowledge of the mechanical behavior of conglomerate under these conditions enables better prediction of geothermal well stability, evaluation of formation failure risks during development, and the design of more effective extraction strategies. Moreover, the findings offer valuable insights for engineering measures in geothermal resource development, such as optimizing well design and blasting techniques.

## Data Availability

All data generated or analyzed during this study are included in this published article.
